# Controlling the angiogenic switch in developing atherosclerotic plaques: Possible targets for therapeutic intervention

**DOI:** 10.1186/2040-2384-1-4

**Published:** 2009-09-21

**Authors:** Mark Slevin, Jerzy Krupinski, Lina Badimon

**Affiliations:** 1 Centro de Investigación Cardiovascular, CSIC-ICCC, Hospital de la Santa Creu i Sant Pau, Barcelona, Spain; 2 School of Biology, Chemistry and Health Science, Manchester Metropolitan University, Manchester, UK; 3 Department of Neurology, Stroke Unit, University Hospital of Bellvitge (HUB), Fundacio IDIBELL, Barcelona, Spain; 4 CIBERobn-Fisiopatología de la Obesidad y Nutrición, Instituto Salud Carlos III, Santiago de Compostela, Spain; 5 School of Medicine, University of Manchester, Oxford Road, UK

## Abstract

Plaque angiogenesis may have an important role in the development of atherosclerosis. Vasa vasorum angiogenesis and medial infiltration provides nutrients to the developing and expanding intima and therefore, may prevent cellular death and contribute to plaque growth and stabilization in early lesions. However in more advanced plaques, inflammatory cell infiltration, and concomitant production of numerous pro-angiogenic cytokines may be responsible for induction of uncontrolled neointimal microvessel proliferation resulting in production of immature and fragile neovessels similar to that seen in tumour development. These could contribute to development of an unstable haemorrhagic rupture-prone environment. Increasing evidence has suggested that the expression of intimal neovessels is directly related to the stage of plaque development, the risk of plaque rupture, and subsequently, the presence of symptomatic disease, the timing of ischemic neurological events and myocardial/cerebral infarction. Despite this, there is conflicting evidence regarding the causal relationship between neovessel expression and plaque thrombosis with some in vivo experimental models suggesting the contrary and as yet, few direct mediators of angiogenesis have been identified and associated with plaque instability in vivo.

In recent years, an increasing number of angiogenic therapeutic targets have been proposed in order to facilitate modulation of neovascularization and its consequences in diseases such as cancer and macular degeneration. A complete knowledge of the mechanisms responsible for initiation of adventitial vessel proliferation, their extension into the intimal regions and possible de-novo synthesis of neovessels following differentiation of bone-marrow-derived stem cells is required in order to contemplate potential single or combinational anti-angiogenic therapies. In this review, we will examine the importance of angiogenesis in complicated plaque development, describe the current knowledge of molecular mechanisms of its initiation and maintenance, and discuss possible future anti-angiogenic therapies to control plaque stability.

## Introduction

According to a World Health Organization Fact Sheet (EURO/03/06) cardiovascular disease (CVD) is the number one killer in Europe, with heart disease and stroke being the major cause of death in all 53 Member States. Figures show that 34,421 (23% of all non-communicable diseases) of Europeans died from CVD in 2005. The report also highlighted the fact that there is approximately a 10-fold difference in premature CVD mortality between Western Europe and countries in Central and Eastern Europe with a higher occurrence of CVD amongst the poor and vulnerable. Although improvements in understanding have helped to reduce the number of Western European dying from CVD and related diseases further advances will require a clearer understanding of the pathobiological mechanisms responsible for the development of stroke, atherosclerosis and myocardial infarction. Approximately 75% of acute coronary events and 60% of symptomatic carotid artery disease are associated with disruption of atherosclerotic plaques [1]. *In 1971, Folkman *[2]*introduced the concept of angiogenesis as a necessity for tumour growth. Its importance in other pathological conditions, including, atherosclerosis, myocardial infarction and stroke was later realized *[3,4]. Angiogenesis is the formation of new blood vessels from a pre-existing vascular network, and is an important phenomenon in physiological situations such as embryonic development and wound healing as well as in pathological conditions like diabetic retinopathy, rheumatoid arthritis, tumor progression and atherosclerosis. Endothelial cells (EC) which line the inside of blood vessels are the target cells of angiogenic regulators. Stimulated EC undergo metabolic modifications associated with the main steps of angiogenesis i.e. production of matrix metalloproteinases that degrade the basement membrane and extracellular matrix (ECM); stimulation of EC migration and proliferation; secretion of collagen and differentiation resulting in sprout formation and ultimately, formation of new blood vessels [3,5].

### Angiogenesis in cardio- and cerebrovascular disease: Evidence for it as a major determinant of plaque growth, instability and rupture

#### Histological characteristics of plaque angiogenesis

In normal arteries, adventitial blood vessels originating from the vasa vasorum penetrate into the vessel wall at regular intervals and bifurcate around the circumference of the artery. They don't extend beyond the outer media, and diffusion of oxygen and nutrients is limited to approximately 100 μm from the vessel lumen [6]. Angiogenesis is a recognized feature of the atherogenic process in both coronary and carotid disease, intimal neovascularization arising frequently following activation and proliferation of the dense network of vessels in the adventitia adjacent to a plaque [7]. New vasa vasorum (VV) have been identified in developing lesions and these invade the intima at specific sites of medial disruption, whilst the intima and media of complicated plaques in coronary atherosclerotic vessels have been shown to be infiltrated with a tumor-like mass of microvessels which are prone to leak [8]. These angiogenic blood vessels have been identified in complicated regions of human aortic lesions following immuno-staining with antibodies to CD105 (endoglin; which binds only to active endothelial cells) and transforming growth factor-beta 1 (TGFβ-1) [9]. Immature neovessels are most often localized in regions of inflammatory/macrophage cell infiltration (and less common in highly calcified or hyalinized plaques) and at the shoulders of thin-cap atheromas prone to rupture [10]. In a study of coronary artery atherogenesis, lesions were examined from coronary arteries from excised hearts of patients. The highest neovessel content was demonstrated in type VI plaques and this was associated with the highest rate of thrombotic episodes [11], whilst other post-mortem studies showed approximate increases in vessel density of twofold in vulnerable plaques and fourfold in disrupted plaques compared with severely obstructed but stable lesions [12]. Similarly, increased angiogenesis and angiogenic gene expression measured using microarrays, together with significantly thinner plaque fibrous caps was associated with symptomatic carotid atherosclerosis [13].

Some earlier studies failed to identify a direct relationship between plaque angiogenesis and instability and direct evidence for the contribution of VV proliferation and/or intimal neovascularization as a cause of plaque rupture is still limited. Part of the reason for this may have been the lack of suitability and similarity between experimental models of atherosclerosis designed using small mammals, which often produce small plaques with limited neovascularization, possibly due to the much smaller diameter of the vessels and media thickness, compared with human vessels and hence many people do not believe they can be used reliably to predict development of unstable lesions in humans [for a review see, [14]]. Several patient studies also demonstrated that plaque haemorrhage in human coronary atherosclerosis was not always a feature of intimal areas with high microvessel density [15], whilst intimal microvessels were only detected in 64% of restenotic human coronary atherosclerotic specimens [16]. However, although the presence of a vascular cell neointimal region is not a prerequisite for unstable plaque development, overwhelming evidence now points to this process as being a key instigator of plaque fragility leading to rupture. Various studies have demonstrated a clear association between VV activation and neointimal formation. In animal models, VV angiogenesis enhanced neointima formation in both the rabbit collar and rat angioplasty models of carotid artery injury, whilst perivascular adenoviral gene transfer of dominant negative FGFR-1 significantly attenuated VV angiogenesis in response to injury [17]. Further evidence is supplied below:

#### Abnormal morphological features and hemorrhagic transformation are features of angiogenic neovessels

**Figure 1:**
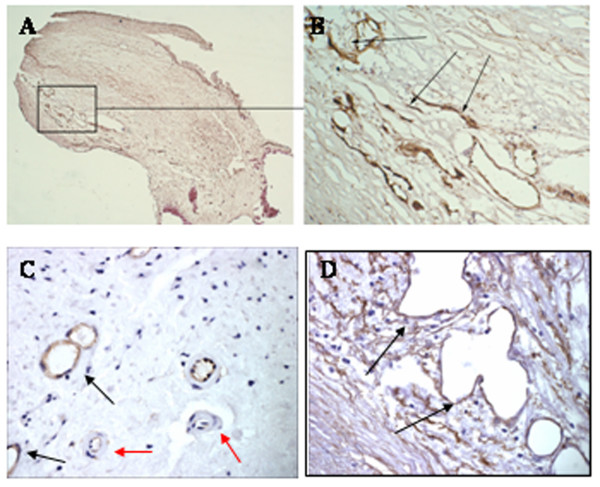
**A-B) CD105 positive neovessels in a grade V carotid plaque from a patient who had undergone endarterectomy**. Grading was based on the classification of Stary [67]; lipid containing plaques with evidence of inflammation and angiogenesis) Only selected microvessels are CD105 positive in these lesions suggesting a dynamic feature of angiogenesis within the plaque architecture. A is × 40 and B × 100. C) Shows expression of immature neointimal vessels composed of only endothelial cell lining and stained positive for CD105 (black arrows), in contrast to more mature vessels negative for CD105 and possessing a smooth muscle cell coating (red arrows) Photomicrographs are × 100. D) Shows morphologically distinct multilobulated abnormally developing vessels again, stained positive for CD105 without smooth muscle cell or pericyte involvement in the neointima of a grade V plaque (× 100).

The irregular nature of blood vessel formation, and its similarity to tumor angiogenesis, suggests that the factors responsible for their growth may be different from those seen during normal wound healing. Newer technological approaches have been able to discriminate between localised micro-environments in individual neointimas using antibodies directed to proteins expressed in actively growing vessels. These studies showed the presence of areas of mature vessel development where haemorrhage was absent as opposed to those containing irregular immature and highly angiogenic vessels where haemorrhage was taking place, and which may help to explain these earlier contadictory findings. For example, Dunmore et al, [18], demonstrated that irregular dysmorphic heamorrhagic vessels without smooth muscle cell coatings, were almost exclusively found in plaques from patients with symptomatic carotid artery disease. Furthermore, these single-layer endothelial tubes co-localized specifically with pockets of VEGF expression providing strong support for the idea that proliferating immature neovessels may be associated with plaque instability and rupture. Similarly, haemorrhagic, leaky blood vessels from unstable carotid plaques proliferate abnormally, and although of relatively large caliber remained immature by virtue of a poor investment with smooth muscle cells and pericytes. These vessels also possessed structural weaknesses with poorly formed EC junctions that may also contribute to instability of the plaque by facilitation of inflammatory cell infiltration and haemorrhagic complications [19], (Figure 1; [20]). In vivo studies showed a strong correlation between areas of increased vascularity, intraplaque haemorrhage and neurological events defined by CT, with subsequent histological staining using anti-CD34 antibodies visualizing EC of the vessels in symptomatic patients following endarterectomy [21].

Intraplaque haemorrhage results in rapid expansion of the plaque necrotic core, due to the fact that red blood cell membranes are a rich source of free cholesterol and phospholipids and the process occurs in association with excessive macrophage infiltration [22]. The size of the necrotic core directly correlates with the risk of plaque rupture. Furthermore, intraplaque haemorrhage and plaque rupture were proportional to neovessel density in coronary atheroma [23]. Adventitial vessels in unstable plaques contain perivascular smooth muscle cells, however, after plaque rupture, the fibrous cap is disrupted with a luminal thrombus and the newer branches of vasa vasorum close to the necrotic core consist almost entirely of a single layer of EC overlying a ruptured, leaky basement membrane, and associated with remnants of red blood cells [24]. Defects are thought to be caused by proteolytic damage from on-going inflammation and release of signalling molecules affecting cell-cell contact.

### Relationship between inflammation and angiogenesis in promotion of plaque development?

The atherosclerotic process is often initiated before adulthood, when cholesterol-containing low-density lipoproteins accumulate in the intima and activate the endothelium. Leukocyte adhesion molecules and chemokines promote recruitment of monocytes and T cells. Monocytes can differentiate into macrophages and up-regulate pattern recognition receptors, including scavenger receptors and toll-like receptors. Scavenger receptors mediate lipoprotein internalization, which leads to foam-cell formation. Toll-like receptors transmit activating signals that lead to the release of cytokines, proteases, and vasoactive molecules, and are considered to be an important link between inflammation and cardiovascular disease [25]. Deficiency of toll-like receptor 4 (TLR-4) protein leads to a reduction in macrophage recruitment in association with reduced cytokine and chemokine levels [26]. T cells in lesions recognize local antigens and mount T helper-1 responses with secretion of pro-inflammatory cytokines that contribute to local inflammation and growth of the plaque [27].

VV density, their proliferation and medial-intimal infiltration, and concurrent adventitial inflammation are strongly associated with advanced lesions [28]. Plaque neovascularization correlated with the extent of inflammation in hypercholesterolemic apoE mice and inhibition of vessel formation reduced macrophage accumulation and plaque progression [22]. Similarly, transfection with murine soluble VEGF-R1 inhibited early inflammation and late neointimal formation, again suggesting that as neovessels are generated, so the inflammatory response is perpetuated in a continuous cycle [29]. Inflammatory infiltrates enhance recruitment of monocytes, secrete matrix metalloproteinases and increase the expression of γ-interferon (from t-lymphocytes) which may weaken the fibrous cap; similarly, they can induce synthesis of angiogenic tissue angiotensin-converting enzyme, growth factors, interleukin-8 and tissue factor [15]. The importance of the inflammatory response was demonstrated in vivo where oral treatment of apoE-/LDL-double knockout mice with the anti-inflammatory compound 3-deazaadenosine prevented lesion formation [30]. A strong correlation has been shown between macrophage infiltration, intraplaque haemorrhage and rupture-prone thin-cap lesions with high microvessel density, whilst these features are not common in calcified or hyalinized human arterial plaques, suggesting a strong link between neovascularization, inflammation and thrombosis [15,31]. The phenotype of plaque neovessels could also be important in determining plaque stability, for example, new vascular networks, and immature vessels with poor integrity and no smooth muscle cell/pericyte coverage would likely act as sites for inflammatory cell infiltration, inflammatory cell leakage and intraplaque haemorrhage respectively [22]. The correlation of focal collections of inflammatory cells with areas of intraplaque neovascularization and haemorrhage, suggests that release of growth factors and cytokines by macrophages and leukocytes may have a key role in modulating the vascularization process [8]. Evidence for the existence of hotspots or "neovascular milieu" was found in lesions from ApoE-/- mice where the density of VV was highly correlated with the presence of inflammatory cells rather than plaque size, whilst deposition of RBC membranes within the necrotic core of plaques also leads to an increase in macrophage infiltration and therefore may further potentiate the inflammatory response [22,23].

Hypercholesterolaemia may be associated with proliferation of VV in coronary and carotid vessels at early stages of plaque development. Williams JK [32] first demonstrated that the presence of atherosclerosis in hypercholesterolemic monkeys induced an increase in blood flow through the VV and plaque regression caused by removal of the high lipid diet reduced the VV concentration and blood flow to the coronary media and intima. High cholesterol levels are associated with increased serum VEGF expression and may cause up-regulation of growth factor receptors on both endothelial and smooth muscle cells [33]. Furthermore, oxidized LDL (ox-LDL) generated in response to pro-oxidative cellular changes can exacerbate the inflammatory response, since engulfment of intact apoptotic cells was reduced in the presence of ox-LDL and in its absence, rapid phagocytosis suppressed macrophage inflammatory cytokine release, suggesting a link between high lipid levels, inflammation and possibly angiogenesis [33]. In vitro studies have demonstrated that stimulation of HUVEC with ox-LDL up-regulates adhesion molecules (including ICAM-1, E-selectin and P-selectin), inflammatory proteins including Il-6, thrombotic factors including tissue factor and remodeling proteins such as MMP-2 and MMP-9, many of which are also stimulators of angiogenesis [34].

Medial and intimal thickening induced by hypercholesterolemia may result in a limited supply of oxygen and nutrients reaching these areas from either the lumen and/or VV, resulting in a hypoxic environment. Since the major outcome of hypoxia is increased vascularization, intraplaque vessels may proliferate in association with this potent stimulus. Hypoxia-inducible factor (HIF-1) is expressed in hypoxic regions of expanding and developing plaques, and directs migration of EC towards the hypoxic environment via direct HIF-1 binding the regulatory gene of VEGF and subsequent induction of VEGF transcription [35]. VEGF is a potent angiogenic growth factor, stimulating EC mitogenesis and blood vessel formation via activation of intracellular signalling intermediates including mitogen-activated protein kinase 1/2 (MAPK1/2) and src. Increased expression of VEGF and its receptors in hypoxic areas, in association with interaction with cell membrane integrins including α_5_β_3_, is one of the main causes of vessel leakiness [36]. Leaky plaque VV have been identified by ultrastructural visualization of defects between endothelial tight, gap and adherens junctions, and VEGF is known to affect junctional adhesion molecule expression, block gap junctional communication between adjacent endothelial cells and disrupt tight junctional communication through a src-dependent pathway [37].

Oxidized phospholipids such as 1-palmitoyl-2-arachidonoyl-*sn*-glycero-3-phosphorylcholine (Ox-PAPC), also prevalent in atherosclerotic lesions, can also up-regulate VEGF expression. In addition, they can regulate leukocyte-endothelial cell interactions and induce expression of inflammatory cytokines from local endothelial cells, monocytes and macrophages [38].

#### In vivo evidence for modified expression of pro/anti-angiogenic molecules in high grade plaques

In the quiescent state, VV of normal arteries provide nutrients to the tunica media whilst the intima is fed by oxygen diffusion from the lumen. The VV remains in a dormant state probably due to the expression and synthesis of anti-angiogenic factors e.g. thrombospondin and endostatin which more than counterbalance the presence of low quantities of pro-angiogenic factors in the micro-environment [39]. However during intimal plaque development, the balance between the angiogenic and anti-angiogenic factors may become altered with increased production of growth factors and cytokines together with a reduction in negative modulators resulting in adventitial vessel angiogenesis at the site proximal to internal vascular damage and plaque growth [40]. **Hence the angiogenic switch shifts to on.**

Key molecules involved in initiation and/or maintenance of the angiogenic process, and which may prove to be potential therapeutic targets of modulation include the angiopoietin-Tie signaling pathways. Ang-1 and -2 are ligands of the endothelial receptor Tie-2. Ang-1 via Tie-2 binding induces formation of stable blood vessels, whereas Ang-2 destabilizes the interaction between EC and their support cells [41]. In a recent study by Post *et al *[42], a positive correlation was observed between the ratio of expression of Ang-1/Ang-2, the macrophage count and mean vessel density in complicated fibroatheromatous carotid human plaques. Ang-2 also correlated with the activity of matrix metalloproteinase-2 (MMP-2) expression suggesting a role in development of unstable plaque microvessels. Tie-2 is a tyrosine kinase receptor expressed predominantly on EC, and which has been shown to activate intracellular signal transduction through Akt and ERK1/2 following ligand binding of angiopoietin-1 resulting in angiogenesis both in vitro and in vivo. In relation to tumour neovascularization, expression of Tie-2 was concentrated in vascular hot-spots at the leading edge of invading tissue [43]. Furthermore, Ang-1 is an anti-inflammatory cytokine which can reduce neovessel leakage and vascular permeability partly via inhibition of cell membrane adhesion molecule expression, whilst an increase in Tie-2 together with increased availability of Ang-1 may enhance neointimal vascularization and promote formation of stable vessels less prone to haemorrhage [44]. Placental growth factor (PIGF), a member of the VEGF family, binds to the Flt-1 receptor, is a mediator of inflammation and is highly expressed in atherosclerosis, correlating with both levels of plaque inflammation and microvascular density in symptomatic patients with carotid plaques [45]. Many other pro-angiogenic factors have been demonstrated to be present in neointimal lesions although direct evidence for their involvement in mediating vascularization in vivo is still lacking and hence they have not been described in this review. Worthy of note, however, is the work of Leroyer *et al *[46] showing that atherosclerotic plaques contained a large number of membrane-shed microparticles originating from the breakdown of apoptotic macrophages. Isolated microparticles were highly angiogenic both in vitro and in vivo and those obtained from symptomatic patients expressed more CD40L and were more potent in mediation of angiogenesis which was blocked in the presence of CD40-neutralising antibodies, suggesting a novel mechanism for stimulation of plaque neovascularization.

Key inhibitors of angiogenesis may be reduced in areas undergoing neovascularization and the balance of pro-angiogenic and anti-angiogenic molecule expression shifts towards blood vessel formation. Thrombospondin-1 (TSP-1) has been implicated in development of vascular diabetic complications and is expressed in developing arterial plaques [47]. Platelet factor IV is expressed by active platelets in plaques [48], whilst increased expression of Collagen XVIII and its proteolytically released endostatin fragment is associated with inhibition of angiogenesis in the vascular wall, and it has been shown that ApoE knockout collagen XVIII minus animals developed significantly more vasa vasorum than wild type [39]. T-cadherin, is involved in maintenance of vascular integrity and growth, is up-regulated in developing atherosclerotic lesions. The effect of T-cadherin on angiogenesis in vivo was evaluated in detail by Rubina et al, [49] using a Matrigel implant model. They demonstrated that T-cadherin over-expression in L929 cells injected in Matrigel inhibited neovascularization of the plug. In vitro, T-cadherin inhibited the directional migration of endothelial cells, capillary growth, and tube formation but had no effect on endothelial cell proliferation, adhesion, or apoptosis.

### Pharmacological inhibition of VV angiogenesis inhibits plaque development and instability in vivo models of atherosclerosis

*In vivo *studies showed that blocking blood vessel formation can significantly reduce plaque size. Moulton *et al*, [50] demonstrated that two potent angiogenesis inhibitors, endostatin and TNP-470 were able to reduce neovessel formation and concomitant aortic plaque growth in apolipoprotein E (ApoE)-deficient mice. Recently, Stefanadis *et al*, [51], showed that treatment of New Zealand rabbits under atherogenic diet, with antibodies specific for VEGF, resulted in significant reduction in neovessel growth and neointimal thickness after four weeks. Similarly, Gossl *et al*, [52] showed that inhibition of VV neovascularization in high cholesterol diet treated domestic swine using thalidomide, significantly reduced neointimal plaque formation and intima-media thickness. The same authors also demonstrated that areas of the coronary vascular wall with low density of VV were most susceptible to microinflammation, hypoxia and oxidative stress making them most likely the starting points of early atherogenesis [53]. Similarly, Drinane et al [54] showed that treatment of LDLR(-/-) ApoB-48-deficient mice with an anti-angiogenic truncated form of plasminogen activator inhibitor (PAI) inhibited VV growth through a mechanism involving FGF-2 and resulted in reduced plaque development. Reduced blood flow to the end branches of the VV as seen in patients with vascular risk factors could lead to inflammation followed by increased permeability, LDL-lipoprotein up-take and macrophage phagocytosis leading to foam cell formation [55]. These results suggest that anti-angiogenic therapy may have beneficial effects in patients by modulating plaque vascularization. Whilst it is important to have normal healthy functional VV, prevention of their proliferation could represent the first stage of therapy against atherosclerotic plaque formation.

#### Identification of a blueprint for the angiogenic switch-future treatment perspectives

Atherosclerosis is a chronic inflammatory disorder, and angiogenesis plays a complex role in atherothrombosis. In addition it is recognized that during plaque development many pro-angiogenic pathways are re-activated and this leads to formation of immature blood vessels prone to rupture. Therefore, inhibition of angiogenesis might be an important target to prevent the development of active, unstable plaque lesions. ***Identification of pathophysiological changes related to angiogenesis may lead in future to design of novel treatments for inhibition of plaque development***.

In vascular plaques, abnormally behaving cells are surrounded by heterogeneous tissue elements, such that the areas of interest/diseased cells may constitute less than 5% of the volume of a tissue biopsy specimen and also may consist of dynamic micro-hotspots which are difficult to analyse in great detail using conventional dissection and non-quantitative global gene or protein microarrays. Hence, techniques such as laser-capture micro-dissection for isolation of specific microvessels in both the VV and intima of evolving lesions could help to identify both the genetic fingerprints associated with blood vessel activation and stimulatory factors associated with the surrounding inflammatory micro-areas in complicated unstable plaque regions. This technique has been used previously successfully by Roy *et al*, [56] who compared global gene expression between blood vessels isolated by laser-capture from normal skin and identified important de-regulated genes including heparin sulphate 6-O-endosulphatase1 (anti-angiogenic) and a reduction in CD24 (angiogenic) in vessels isolated from within chronic wounded tissue. Our recent un-published work employing Real-time PCr, TaqMan angiogenesis-directed microarrays has demonstrated concurrent over-expression of NOTCH-3/DLL4 and Ang-1/Tie-2 proteins as well as KDR, HBEGF and RAGE in the same microvessels from micro-dissected angiogenic (CD105-positive) vessels from unstable human endarterectomy specimens, whilst these proteins were weakly expressed or not expressed in the same lesion/section in an area containing stable inactive vessels, suggesting a complex but specifically localized activation and signaling cascade is involved in mediating vascularization in growing neointimal lesions. This suggests that actively growing neovessels are re-expressing these embryologically derived genes which have been shown to regulate formation of tip cells during angiogenesis, and therefore may form part of an attempt to regulate formation of stable vessels which could be less prone to leakage [57]. Since inhibition of Notch signalling has been shown to increase the total number of vessels in solid tumour models but reduce the patency and vascular function of the remaining vessels (resulting in "chaotic" vessels), it may have similar effects on neointimal vessels and supplementation could be considered as part of a therapeutic programme of normalization and stabilization of existing vasculature promoting plaque stability. Some of the current research is directed to **endothelial progenitor cells**. They appear to be mobilized from the bone marrow by angiogenic factors to the adventitial space, and recent studies have shown that progenitor cells in the adventitia can contribute to progression of atherosclerosis, and EPC concentrations were significantly associated with severity and presence of multivessel coronary artery disease [58]. It is possible that these progenitor cells may be involved in development of new vasa as well as neointimal leaky vessels and therefore future therapeutics might also consider methods to restrict their differentiation in angiogenic microenvironments of developing plaques.

***Knowledge of which factors are expressed and at what times during plaque development may help in the design of a new therapeutic approach to target multiple mediators at different time windows during plaque development***.

**Figure 2:**
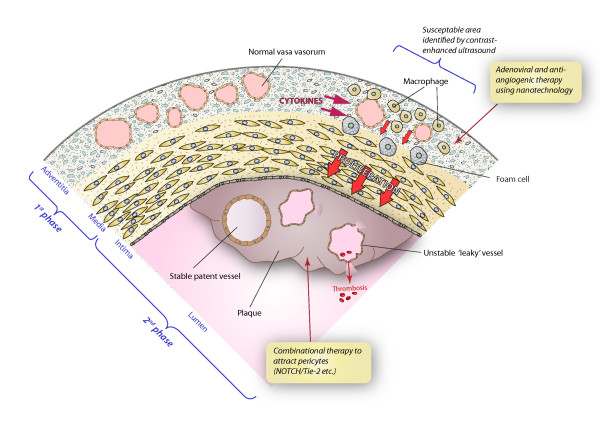
**Hypothetical representation of a micro-area of a major artery showing vasa vasorum and potential hypoxic micro-environment responsible for cellular activation, endothelial cell proliferation and ultimately plaque development**. Phase 1 treatment may involve prevention of vasa vasorum proliferation by administration of targeted siRNA to block relevant gene expression. In patients with existing high grade atherosclerotic plaques, the neointima often presents with microvessels of differing levels of maturity and stability/patency. Phase 2 treatments would be designed to induce stabilization of vessels liable to rupture and bleed by administrating a mixture of factors to induce maturation of fragile vessels. Vascularization in atherosclerosis represents a complex interaction between multiple molecules, manipulation of which could benefit patients by slowing down the process of atherosclerosis and stabilizing existing plaques.

Accurate identification of VV susceptible to development of hypoxic microenvirnments is likely to become a possibility in the next few years with the development of contrast-enhanced and targeted ultrasound imaging (Magnoni *et al*, 2009; Granada *et al*, 2008) [59,60]. Adenoviral delivery of genes, directed against angiogenic molecules, to the perivascular surface of these VV in order to inhibit their proliferation would represent a first line of attack, whilst pharmacological treatments to reduce stem cell differentiation and/or reduce local inflammation and cytokine release, appears to be the most likely avenue for future combinational therapies [[61]; Figure 2]. Weak neovascular beds in plaque intima as well as activated adventitial blood vessels are potential targets for molecular imaging and targeted drug therapy, however, the majority of potential imaging and therapeutic agents have been unsuccessful because of their limited capacity to reach and remain stably within the target tissue or cells in vivo. The idea of using peri-vascular combinational gene therapy for inhibition of VV activation was mentioned in an earlier section. Advantages of this outside-in mechanism is that the integrity of the vessel wall would remain intact and high concentrations of gene delivery vectors can be retained in the local environment, producing minimal inflammatory reaction, when compared with current systemic therapies [62].

Molecular imaging for the visualization of vulnerable atherosclerotic plaques may be useful in detecting patients at risk of thrombosis and by recognition of individual cellular and molecular characteristics, provide the potential for offering personalized medical treatment. For example, when large numbers of paramagnetic gadolinium complexes (>50,000) are incorporated onto emulsion particles, the signal enhancement for each binding site is magnified dramatically compared with conventional contrast reagents [[63] and references therein]. Neovessels in coronary plaques of cholesterol-fed apoE mice have been imaged using vascular cell adhesion molecule-1 peptide sequence (VHSPNKK) bound to cross-linked magneto-fluorescent super-paramagnetic iron oxide (CLIO) particles. The particles were shown to selectively bind to aortic plaque vasculature 24 h after injection using MRI and ex-vivo MR and corresponded with histology and fluorescent analysis of tissue samples performed after euthanasia, suggesting potential diagnostic and therapeutic applications [64]. In humans, contrast-enhanced carotid ultrasound imaging was able to identify intra-plaque neovascularization which correlated with later histological findings employing the use of antibodies directed to CD31 and CD34 for identification of the vessels [65]. Similarly, α_5_β_3 _integrin targeted perfluorocarbon nanoparticles have been successfully used to detect neovasculature in a rabbit model of aortic valve disease suggesting a potential application in humans. In addition, the same technology could be used to home to angiogenic areas or microenvironments, and release targeted knockout genes in order to block VV proliferation, and thereby reduce neointimal expansion or to inhibit neointimal angiogenesis directly or maturate existing fragile rupture-prone vessels. It is important to remember that bioactive molecules of the extracellular matrix including proteoglycans such as hyaluronan whose turnover as regulated by growth factors such as transforming growth factor-β and proteases including matrix metalloproteinases, would also be considered potential therapeutic targets for controlling the angiogenic switch in specific susceptible microenvironments [66]. Anti-angiogenic agents have previously been shown to "prune" tumour vessels and normalize the structure of remaining vessels and therefore might help to stabilize rupture-prone plaques via a similar mechanism [67]. In relation to the process of angiogenesis, fragile endothelial cells of angiogenic neovessels express specific markers such as α_5_β_3 _integrin, CD105 receptors VEGF-R2 and/or apelin [68], which could be used in this targeting protocol.

## Conclusion

Current imaging methodologies may be used in conjunction with emerging therapies based on the use of angiogenesis-targeted nanoparticles which will allow both the identification and tracking of drugs and also prove to be more effective for the delivery of drugs to target sites. The preferential aim would be to prevent initial development of neointimal vascular sites by blocking adventitial blood vessel activation in patients with low grade plaques might represent 1) future imaging targets able to identify patients developing unstable plaque regions susceptible to rupture, and 2) prevent or slow down development of atheroma thereby improving treatment and survival rates of patients with a history of development of myocardial infarction or ischaemic stroke.

## Competing interests

The authors declare that they have no competing interests.

## Authors' contributions

MS, JK and LB were involved in preparation of the manuscript. All the authors read and approved the final manuscript.
